# Supplementary material for: Two-Dimensional Heterostructure of PPy/CNT–E. coli for High-Performance Supercapacitor Electrodes
Source: Materials (Basel). 2022 Aug 23;15(17):5804. doi: 10.3390/ma15175804 (PMC9457316; doi:10.3390/ma15175804)
Supplement: Supplementary file 1 [file materials-15-05804-s001.zip › materials-1814715-supplementary.pdf]

Supplementary Materials

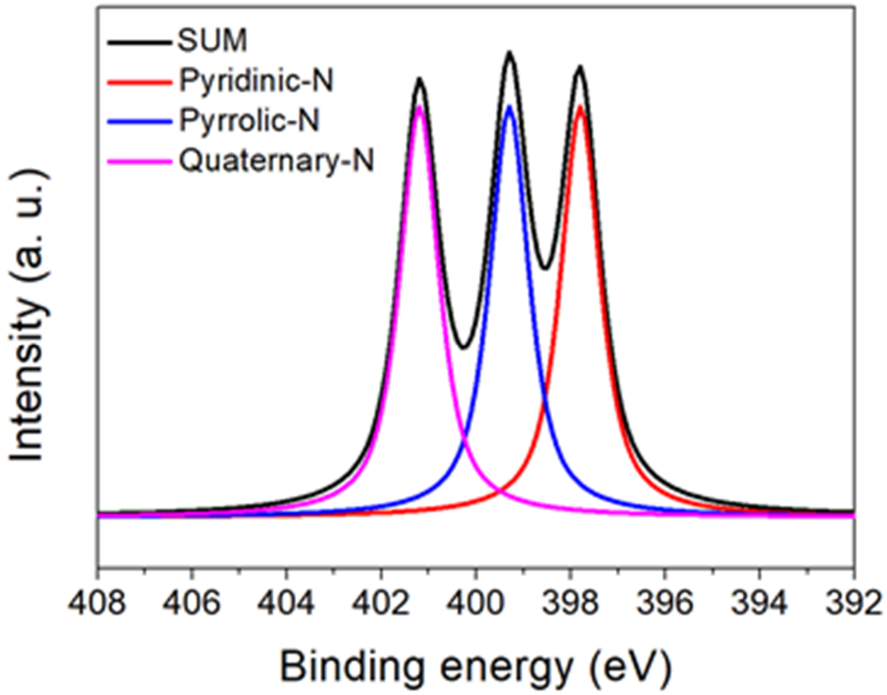

Figure S1. XPS survey of (a) E. *coli* bacteria.

Table S1. Contents of nitrogen species in E. *coli* bacteria.

	**Pyridinic-N**	**Pyrrolic-N**	**Quaternary-N**
E. coli bacteria	33.3	33.3	33.3
